# Supplementary material for: Pyruvate Dehydrogenase Kinase 1 inhibition mediated oxidative phosphorylation enhancement in cartilage promotes osteoarthritis progression
Source: BMC Musculoskelet Disord. 2023 Jul 20;24:597. doi: 10.1186/s12891-023-06585-6 (PMC10357736; doi:10.1186/s12891-023-06585-6)
Supplement: Supplementary file 1 — Additional file 1. [file 12891_2023_6585_MOESM1_ESM.zip › MF.Gsea.1653627316242/GOMF_CARBOHYDRATE_KINASE_ACTIVITY.html]

Details for gene set GOMF\_CARBOHYDRATE\_KINASE\_ACTIVITY[GSEA]

|  || Dataset | 55457\_collapsed\_to\_symbols.cls.cls #OA\_versus\_normal.cls.cls #OA\_versus\_normal\_repos |
| Phenotype | cls.cls#OA\_versus\_normal\_repos |
| Upregulated in class | OA |
| GeneSet | GOMF\_CARBOHYDRATE\_KINASE\_ACTIVITY |
| Enrichment Score (ES) | 0.3452234 |
| Normalized Enrichment Score (NES) | 1.1601605 |
| Nominal p-value | 0.20990565 |
| FDR q-value | 0.60108435 |
| FWER p-Value | 1.0 |
Table: GSEA Results Summary

  

Fig 1: Enrichment plot: GOMF\_CARBOHYDRATE\_KINASE\_ACTIVITY      
 Profile of the Running ES Score & Positions of GeneSet Members on the Rank Ordered List

  

| SYMBOL | TITLE | RANK IN GENE LIST | RANK METRIC SCORE | RUNNING ES | CORE ENRICHMENT || 1 | FGGY | FGGY carbohydrate kinase domain containing [Source:HGNC Symbol;Acc:HGNC:25610] | 36 | 1.018 | 0.1401 | Yes |
| 2 | RBKS | ribokinase [Source:HGNC Symbol;Acc:HGNC:30325] | 225 | 0.802 | 0.2381 | Yes |
| 3 | NAGK | N-acetylglucosamine kinase [Source:HGNC Symbol;Acc:HGNC:17174] | 256 | 0.780 | 0.3452 | Yes |
| 4 | PFKM | "phosphofructokinase, muscle [Source:HGNC Symbol;Acc:HGNC:8877]" | 2260 | 0.331 | 0.2362 | No |
| 5 | KHK | ketohexokinase [Source:HGNC Symbol;Acc:HGNC:6315] | 2809 | 0.269 | 0.2314 | No |
| 6 | PFKFB2 | "6-phosphofructo-2-kinase/fructose-2,6-biphosphatase 2 [Source:HGNC Symbol;Acc:HGNC:8873]" | 3547 | 0.198 | 0.2020 | No |
| 7 | HK3 | hexokinase 3 [Source:HGNC Symbol;Acc:HGNC:4925] | 3792 | 0.178 | 0.2079 | No |
| 8 | POMK | protein O-mannose kinase [Source:HGNC Symbol;Acc:HGNC:26267] | 4680 | 0.098 | 0.1528 | No |
| 9 | GALK2 | galactokinase 2 [Source:HGNC Symbol;Acc:HGNC:4119] | 4945 | 0.076 | 0.1430 | No |
| 10 | PFKP | "phosphofructokinase, platelet [Source:HGNC Symbol;Acc:HGNC:8878]" | 5500 | 0.032 | 0.1045 | No |
| 11 | PFKFB4 | "6-phosphofructo-2-kinase/fructose-2,6-biphosphatase 4 [Source:HGNC Symbol;Acc:HGNC:8875]" | 5534 | 0.030 | 0.1062 | No |
| 12 | PFKL | "phosphofructokinase, liver type [Source:HGNC Symbol;Acc:HGNC:8876]" | 5632 | 0.021 | 0.1016 | No |
| 13 | GALK1 | galactokinase 1 [Source:HGNC Symbol;Acc:HGNC:4118] | 5785 | 0.009 | 0.0911 | No |
| 14 | HK1 | hexokinase 1 [Source:HGNC Symbol;Acc:HGNC:4922] | 6728 | -0.058 | 0.0261 | No |
| 15 | XYLB | xylulokinase [Source:HGNC Symbol;Acc:HGNC:12839] | 7054 | -0.084 | 0.0126 | No |
| 16 | HK2 | hexokinase 2 [Source:HGNC Symbol;Acc:HGNC:4923] | 10030 | -0.325 | -0.1728 | No |
| 17 | GCK | glucokinase [Source:HGNC Symbol;Acc:HGNC:4195] | 10137 | -0.335 | -0.1341 | No |
| 18 | HKDC1 | hexokinase domain containing 1 [Source:HGNC Symbol;Acc:HGNC:23302] | 11172 | -0.454 | -0.1507 | No |
| 19 | PFKFB1 | "6-phosphofructo-2-kinase/fructose-2,6-biphosphatase 1 [Source:HGNC Symbol;Acc:HGNC:8872]" | 11840 | -0.557 | -0.1243 | No |
| 20 | PFKFB3 | "6-phosphofructo-2-kinase/fructose-2,6-biphosphatase 3 [Source:HGNC Symbol;Acc:HGNC:8874]" | 12388 | -0.704 | -0.0680 | No |
| 21 | GNE | glucosamine (UDP-N-acetyl)-2-epimerase/N-acetylmannosamine kinase [Source:HGNC Symbol;Acc:HGNC:23657] | 12538 | -0.767 | 0.0281 | No |
Table: GSEA details [plain text format]

  

Fig 2: GOMF\_CARBOHYDRATE\_KINASE\_ACTIVITY      
 Blue-Pink O' Gram in the Space of the Analyzed GeneSet

  

Fig 3: GOMF\_CARBOHYDRATE\_KINASE\_ACTIVITY: Random ES distribution      
 Gene set null distribution of ES for **GOMF\_CARBOHYDRATE\_KINASE\_ACTIVITY**
